# 

**Published:** 1879-05

**Authors:** 


*CONTENTS.*

| | Page | | Page |
|---|---|---|---|
| Traveling for Health | 803 | Idleness | 821 |
| Mind and Charcoal | 810 | The Noble Horse | 821 |
| Facts for the Natives | 815 | Ice Houses | 823 |
| An Afflicted Man | 816 | A Sad Reflection | 825 |
| Ice | 816 | Henry Clay | 826 |
| Shade Trees | 817 | Newspapers | 827 |
| Barns | 817 | Draw Them Up | 828 |
| The Stable | 818 | The Cherished Flower | 829 |
| Water Pipes | 820 | Sanitary | 830 |

